# Rethinking participation: design studios for health as a scalable co-design framework for health equity

**DOI:** 10.3389/fpubh.2025.1681798

**Published:** 2026-01-05

**Authors:** Maissa Khatib, Rushabh Shah, Ehiremen Azugbene, Matthew Paul Buman

**Affiliations:** College of Health Solutions, Arizona State University, Phoenix, AZ, United States

**Keywords:** co-design, participatory research, community engagement, academic–community partnership, citizen science, user-centered health solutions, underserved populations, health equity

## Abstract

**Introduction:**

The Design Studios for Health (DSH) framework is a participatory, partnership-driven model designed to foster equitable collaboration between community members and researchers to address diverse health priorities. This study evaluates outcomes from 11 DSH sessions conducted over 2 years with historically underserved communities, focusing on key indicators of engagement: collaboration, facilitator performance, participant willingness to support each other, group cohesion, and trust. Grounded in design thinking and community-based participatory research (CBPR), the DSH model uses an iterative structure of 2–3 sessions per health topic to support sustained, meaningful engagement. DSH fosters shared ownership, multidirectional learning, and culturally responsive solution development.

**Methods:**

We conducted 11 DSH sessions with historically underserved communities over two years. Mixed-methods data were collected through post-session surveys, open-ended responses, and qualitative debriefs. Quantitative metrics assessed collaboration, facilitator performance, participant willingness to support others, cohesion, and trust. Qualitative analyses explored participant experiences, perceived value, and the role of iterative engagement in fostering co-creation.

**Results:**

Later DSH sessions demonstrated improvements in collaboration, group cohesion, and trust compared to earlier studios. Participants described DSH as a safe, empowering space that promoted deep listening, mutual support, and shared ownership. Iterative engagement allowed community members to refine ideas, elevate cultural insight, and contribute meaningfully to intervention development. A single-session studio on Women’s Brain Health illustrated the model’s adaptability across varied health topics.

**Conclusion:**

DSH functions as a scalable co-design framework that strengthens academic–community partnerships, builds trust, and supports community-led innovation. By centering lived experience and embedding iterative feedback loops, the model offers a practical, equity-oriented approach for co-creating culturally relevant health solutions with underserved populations.

## 1 Introduction

### 1.1 Persistent health inequities in context

Health disparities remain among the most entrenched public health challenges in the United States, disproportionately affecting underserved and historically marginalized populations (1). Black, Latinx, and Indigenous communities experience markedly higher rates of hypertension, diabetes, and obesity and face reduced access to healthcare, education, and economic opportunity (2, 3). These inequities stem from long-standing structural racism, historical disinvestment, and policy neglect shaping the social determinants of health (4, 5). In Arizona, these patterns are especially pronounced. Between January 2020 and July 2022, the Arizona state recorded the nation’s highest age-adjusted COVID-19 mortality—581 deaths per 100,000 residents (6, 7). Latinx Arizonans, particularly monolingual Spanish speakers and uninsured individuals, experienced disproportionate losses and unmet social and medical needs (8). Mental health treatment gaps persist as well: only 39% of Black adults receive care, compared to 52% of White adults (9). These disparities highlight how pandemic stressors compound existing inequities among Black, Indigenous, Latinx, refugee, immigrant, rural, and low-income communities statewide.

### 1.2 Reassessing research practice at PHRI

Recognizing these realities, Arizona State University’s (ASU) Precision Health Research Initiative (PHRI) reassessed its research practices and identified two major gaps: (a) exclusion of community voices from priority setting, and (b) data deficiencies that render marginalized groups functionally invisible (10). In collaboration with ASU Lincoln Center for Applied Ethics and local organizations, PHRI launched an inclusive co-design process enabling community partners to shape project goals, research questions, and evaluation metrics. Regular planning meetings and DSH sessions ensure that solutions remain ethical, contextually grounded, and responsive to Arizona’s diverse populations. Failure to address these inequities perpetuates poor health, economic instability, and eroded trust in health systems (11). The Design Studios for Health (DSH) framework was developed to counter these cycles by embedding community leadership across all stages—from conception through dissemination—to advance equitable, sustainable outcomes.

### 1.3 From traditional engagement to iterative co-design

Even well-intentioned community-engaged research can falter. Models such as community-based participatory research (CBPR) and community-engaged research (CEnR) have advanced equity through shared power and long-term partnerships but often operate on academic timelines that privilege research outputs over community priorities (12–14). These structures can struggle with real-time adaptability and sustained shared decision-making, occasionally reproducing the very power imbalances they aim to dismantle. A related model, the Community Engagement Studio (CES), provides structured stakeholder input to improve research design and implementation (15, 16). While effective, CES tends to function as a one-time consultation rather than a sustained, iterative partnership. In contrast, DSH emphasizes repeated engagement across stages, building rapport, trust, and shared decision-making as design evolves. This approach extends CES’s strengths by layering iterative co-design and ongoing stakeholder roles throughout implementation and evaluation.

### 1.4 The DSH framework: equity, empathy, and adaptability

The Design Studios for Health (DSH) framework weaves equity, empathy, and adaptability into every stage of collaboration, creating a dynamic space for co-design and community leadership. Equity is at the core of the framework, operationalized through shared leadership between community and academic partners. This structure ensures that lived experience carries equal weight alongside disciplinary expertise, positioning community members as co-creators rather than subjects of research—an approach aligned with recent participatory frameworks that emphasize community-led health design (17). Building on this foundation, empathy guides how participants engage and make meaning together. Through practices such as deep listening, community check-ins, and transparent reporting, facilitators create an environment where participants feel heard and affirmed before moving toward collective solutions (18). Finally, adaptability enables DSH to remain responsive to community rhythms and priorities. Its multi-session format allows insights from earlier studios to shape subsequent agendas, ensuring that the process evolves with participants’ feedback and aligns with community timelines rather than academic milestones (19). Together, these principles cultivate a participatory structure that centers trust, shared ownership, and ongoing learning—essential elements for advancing equity in health research and practice.

### 1.5 Integrating CBPR and design thinking

CBPR has been instrumental in advancing health equity but is often limited in flexibility and real-time design capacity (13, 14, 58). Conversely, design thinking brings creativity and iteration to healthcare redesign but typically centers institutional or provider perspectives (18, 20, 21). DSH bridges these traditions by embedding design thinking’s cycles of divergence and convergence within CBPR’s equity-centered engagement practices (22). This integration operationalizes trust, co-learning, and cultural humility within a structured, replicable studio model that centers community leadership and lived experience. Much of the design thinking literature emphasizes provider-led service redesign within clinical settings (20, 21). In contrast, DSH situates design thinking within a community-facing, equity-centered context—foregrounding lived experience, cultural insight, and co-leadership in shaping health interventions. Related initiatives, such as the Mayo Clinic Center for Innovation (21), the Health Design Lab at Thomas Jefferson University (23), and the Health Design Studio at the University of Waterloo (24), highlight the potential of studio methods in health contexts. DSH extends this work by embedding iterative design within the participatory and ethical foundations of CBPR, advancing a model in which community members act as co-designers and decision-makers (46, 52, 53, 56).

### 1.6 Positioning DSH within the studio tradition

Beyond CBPR or CEnR, DSH is distinct from other studio models in health and design education. Architectural and design studios often emphasize creativity and prototyping but rarely incorporate equity or community voice (25–28). Health design studios have been used to reimagine hospital spaces or workflows (20, 29, 30) and to translate design-thinking principles into architectural practice within healthcare contexts (31), but they generally center institutional perspectives.

DSH extends these traditions by positioning marginalized populations as co-designers and co-leaders, embedding trust-building as a core outcome, and structuring multiple touchpoints over time. By countering extractive practices and rigid timelines emphasizing ethical accountability in collaborative design (32), DSH offers a replicable, equity-centered innovation for participatory public health research. It aligns with a growing movement to center community voices in the co-design of health interventions and to challenge conventional research paradigms through sustained, trust-based partnerships (47–51, 57).

### 1.7 Study aim

This mixed-methods study analyzes 11 DSH sessions, integrating quantitative survey data with qualitative insights from debriefs and open-ended responses. The objectives are to:

1. Describe the Design Studios for Health approach and its implementation, and
2. Present initial findings from its application with diverse Arizona communities.

In doing so, we demonstrate how DSH functions as a flexible and effective model for building trust, fostering co-learning and co-creation, and advancing health equity through genuinely collaborative public health research.

## 2 Materials and methods

### 2.1 Setting and context

**Figure 1:**
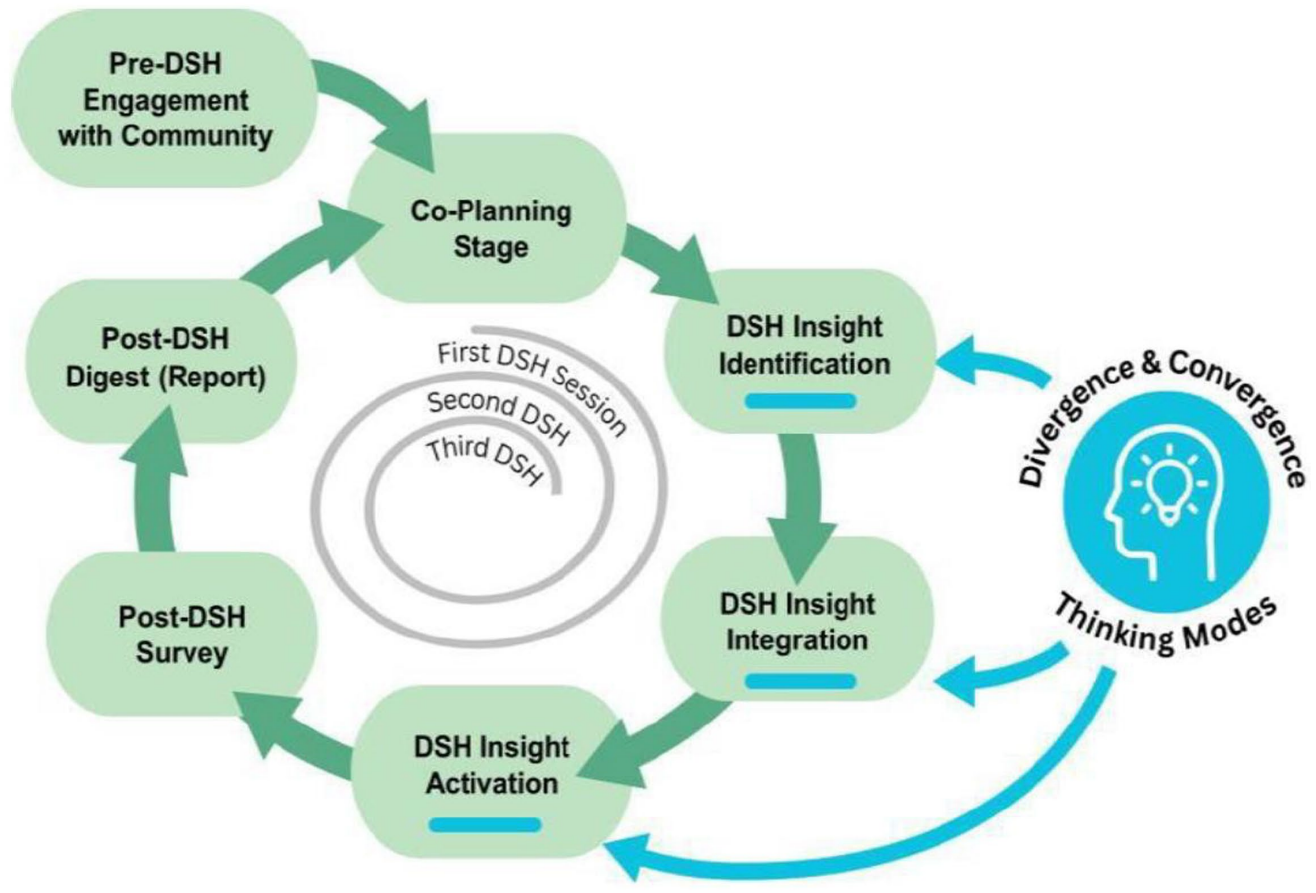
Design studio for health process.

The DSH framework was developed to address persistent challenges in creating sustainable and equitable community-academic partnerships. Rooted in ongoing Arizona-based community engagement efforts led by Arizona State University (ASU), DSH responds to barriers repeatedly identified by community members from Latino/Hispanic, Black, Indigenous, and immigrant/refugee communities. These community partners emphasized deep-seated mistrust in research, trauma related to extractive engagement, and a lack of alignment between academic priorities and community-defined needs. Rather than operating as one-time consultations, DSH sessions unfold as a series of iterative gatherings centered on community-defined health needs (10) such as food security and refugee mental health. Each session is designed to promote multidirectional learning, elevate lived experience as expertise, and generate actionable insights. Concerns that shaped the design included the limitations of short-term, top-down engagement strategies and the misalignment between academic timelines and community priorities (See Figure 1). Each DSH session was a structured, two-hour session facilitated by three core roles:

- A community host who welcomed participants and framed the session around community values
- A facilitator who guided the flow of conversation and ensured inclusivity, and
- A researcher who documented insights and supported follow-up.

Figure 1 illustrates the DSH process. The model begins with pre-DSH engagement with the community, where partners help frame priorities and ensure cultural relevance. This is followed by a co-planning stage, during which community hosts and academic partners jointly shape session agendas, roles, and facilitation strategies. Each DSH session then unfolds in three design thinking–informed phases. In the Insight Identification phase, participants engage in divergent thinking to surface concerns, needs, and potential solutions. During Insight Integration, ideas are synthesized and prioritized through convergent dialogue, resulting in shared understanding and agreement on next steps. Finally, the Insight Activation phase translates these priorities into actionable strategies for intervention design, advocacy, or follow-up research. After each session, a post-DSH survey captures participant feedback, and a digest (report) is shared back with the group to document themes, maintain transparency, and inform the next iteration. This cyclical structure emphasizes adaptability, as each studio feeds into subsequent sessions, allowing the process to evolve in response to participants’ lived experiences and shifting priorities. Over the two-year study period, a total of 11 DSH sessions were conducted across multiple health priorities. Follow-up sessions for each research focus area were held every three to 4 weeks to allow time for reflection, relationship-building, and planning. The academic–community partnership topic required four sessions, as it involved multiple stakeholder groups and addressed complex issues of trust, role clarity, and sustainability that benefited from extended dialogue. In contrast, the menopausal women’s brain health topic was addressed in a single session focused on testing and refining a prototype application that had been developed in earlier phases of work. This structure reflected the iterative nature of DSH: insights generated in one session were intentionally carried forward into the next, enabling participants to refine priorities, revisit emerging themes, and co-develop increasingly actionable strategies over time.

### 2.2 Sample

**Figure 2:**
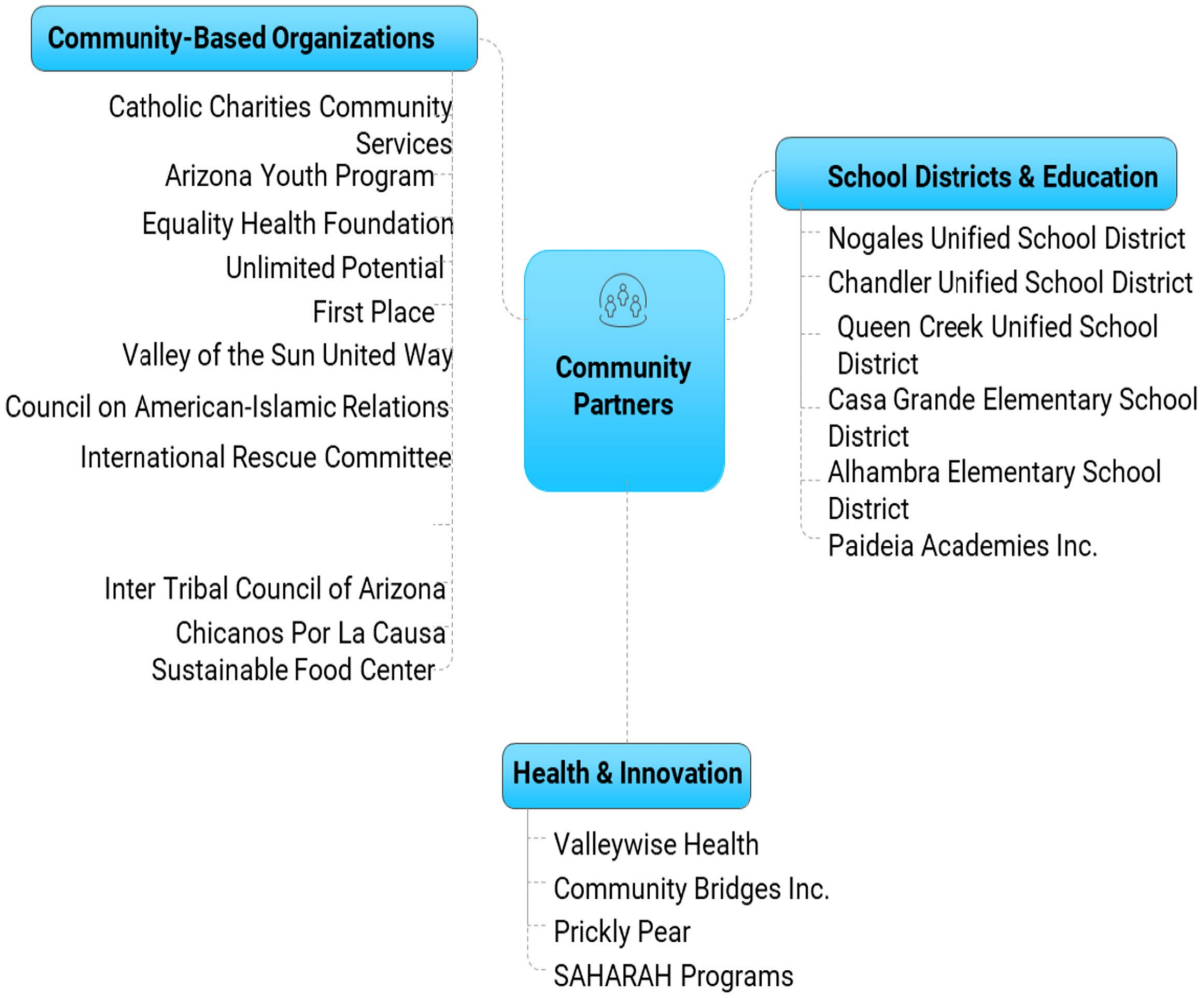
Community partners.

Over the course of 2 years, a total of 11 DSH sessions were conducted in the Phoenix metropolitan area and Austin, Texas. These sessions engaged 90 community members, 15 representatives from community health organizations, and 10 academic researchers (see Table 1). Participants represented underserved and historically marginalized populations navigating health challenges such as limited access to care, food insecurity, and chronic disease. Community partners (see Figure 2) brought experiential insights, while academic partners contributed disciplinary expertise from public health, nutrition, behavioral science, and medicine. This diverse mix fostered the bidirectional knowledge exchange and shared leadership foundational to the DSH framework, creating space for authentic, community-rooted problem-solving.

**Table 1:** Participants’ characteristics.

Group	*N*	Mean age	SD	Education level
Academic Researchers				
Male	3	49	5.29	PhD
Female	7	53	12.38	PhD
Community Members Male				
Male	34	46	11.67	3 High School Diploma, 5 Associate Degrees; 20 Bachelor’s; 4 Mater’s; 2 Doctorate
Female	55	49	11.13	10 High School Diplomas, 12 Associate Degrees; 20 Bachelor’s; 11 Mater’s; 2 Doctorate
Community Health Org Reps				
Male	6	51	14.4	3 Bachelor’s; 2 Master’s; 1 Doctorate
Female	9	46.4	9.9	4 Bachelor’s; 4 Master’s; 1 Doctorate

### 2.3 Data collection

Recruitment strategies were tailored to the two primary participant groups. Community members were recruited through purposive outreach led by trusted community partners, word-of-mouth within neighborhood networks, announcements shared on community-focused Facebook pages, and Slack channels connected to local organizations. This approach prioritized accessibility and cultural relevance, ensuring participation from individuals with lived experience of the health issues under discussion. Academic participants, in contrast, were invited through targeted emails distributed via Arizona State University faculty listservs, departmental announcements, and direct outreach to researchers affiliated with the College of Health Solutions. This dual strategy enabled balanced representation of community expertise and academic perspectives within each DSH session. We used a mixed-methods approach to evaluate the DSH process, incorporating both quantitative and qualitative data to assess participant experiences and the DSH framework impact. After each session, participants were invited to complete a short survey via Qualtrics. The survey included:

- Ten demographic questions,
- Five Likert-scale items measuring perceptions of trust, collaboration, and shared decision-making, and
- Four open-ended questions exploring participants’ experiences, suggestions, and insights.

Likert-scale responses were analysed using descriptive statistics (frequencies, means, and standard deviations). Open-ended responses were analysed using constant comparative analysis, an inductive coding method that allowed for the identification of patterns across studios and over time. Codes were developed iteratively and refined through team discussion to ensure consistency and thematic clarity. Survey items were adapted from validated tools widely used in community-engaged research, including: The Engage for Equity Community Engagement Survey (33), which evaluates partnership quality, shared goals, trust, and sustainability; and The Community Research Collaboration Partnership Assessment Tool (34), which assesses communication, role clarity, and collaborative readiness. These tools provided a reliable foundation while allowing for contextual customization based on participant feedback and evolving needs. The iterative nature of data collection and refinement mirrored the studio process itself, ensuring that evaluation was grounded in community-centered, equity-driven practices. Ethical considerations were reviewed by the Arizona State University Institutional Review Board, which determined this project to be “not human subjects research” (IRB ID: STUDY00017441; March 14, 2023). Although formal approval and signed consent were not required, all participants received information about the sessions, were assured of confidentiality, and chose to participate voluntarily—providing implied consent through their involvement.

### 2.4 Data analysis

The DSH sessions were evaluated using both quantitative and qualitative approaches to provide a comprehensive understanding of participants’ experiences and the overall performance of the DSH model. Each session offered participants a collaborative space to interact with peers, facilitators, and the host, whether in online or in-person settings, to discuss the session’s focus topics. This participatory learning structure mirrors pedagogical models of inverted studios that prioritize active collaboration and reflection (35). Following each session, participants were invited to complete a survey administered via Qualtrics. The survey captured demographic data and participants’ perceptions of their DSH experience.

#### 2.4.1 Quantitative data analysis

To ensure consistency in data collection and to track the performance of the DSH framework over time, the survey included a standardized set of questions divided into three categories: demographic questions, Likert scale questions, and open-ended responses. Specifically, the survey comprised 10 demographic items, 5 Likert scale questions, and 4 open-ended questions. Descriptive analyses, including calculations of means, standard deviations, and frequencies, were conducted to analyze the quantitative responses. Metrics collected included levels of collaboration, group cohesion, and trust, each measured on a 0–5 scale. The survey measures were designed to assess key aspects of the DSH experience, including Collaboration, Facilitator Performance, Participants’ Willingness to Help Each Other, Group Cohesion, and Trust. These metrics provided valuable insights into the effectiveness of the DSH sessions over time, highlighting strengths and identifying areas for improvement, consistent with evaluation tools commonly used in design and engineering teams to measure collaborative performance (36).

Open-ended responses were summarized to capture participants’ suggestions for enhancements, highlight useful components of the sessions, and explore whether participation influenced their ideas or perceptions. A systematic constant comparison method was employed to analyze the responses across all DSH sessions. This approach allowed for the identification of recurring themes and patterns, facilitating a nuanced interpretation of both quantitative trends and qualitative insights. By juxtaposing responses across sessions, we identified areas of consistency and change, enabling a dynamic understanding of participant feedback.

#### 2.4.2 Qualitative data analysis

Qualitative data were generated from notes taken in real time during each DSH session and from short analytic summaries developed immediately afterward. During sessions, a researcher documented participant interactions, tone, and emerging ideas to complement structured survey data and capture nuances of engagement that might not appear in open-ended survey responses. After each session, brief reflective notes were written to consolidate initial impressions, highlight salient insights, and identify areas for follow-up in subsequent studios. These reflections preserved contextual understanding and informed facilitation strategies rather than introducing new data. Of the eleven DSH sessions analyzed, five were conducted virtually via Zoom and six were held in person, reflecting trends in virtual design studio models that enhance accessibility and collaboration in distributed settings (37). All sessions were audio-recorded using Zoom with AI-generated transcription enabled. Each transcript was carefully reviewed against the corresponding recording to verify accuracy and correct any transcription errors. All transcripts and field notes were coded inductively using the constant comparative method (38, 39) to identify recurring patterns, contrasts across sessions, and emergent themes. This approach was selected for its capacity to allow findings to emerge directly from participants’ language and experiences while supporting iterative comparison across sessions. Its cyclical process of reflection and refinement aligns with the participatory and equity-centered nature of the DSH framework, enabling an analysis that remains grounded in community perspectives and responsive to evolving group dynamics. Through this approach, the qualitative data provided rich insight into how the DSH process fostered collaboration, trust-building, and shared decision-making across diverse community contexts.

## 3 Results

### 3.1 Quantitative results

**Figure 3:**
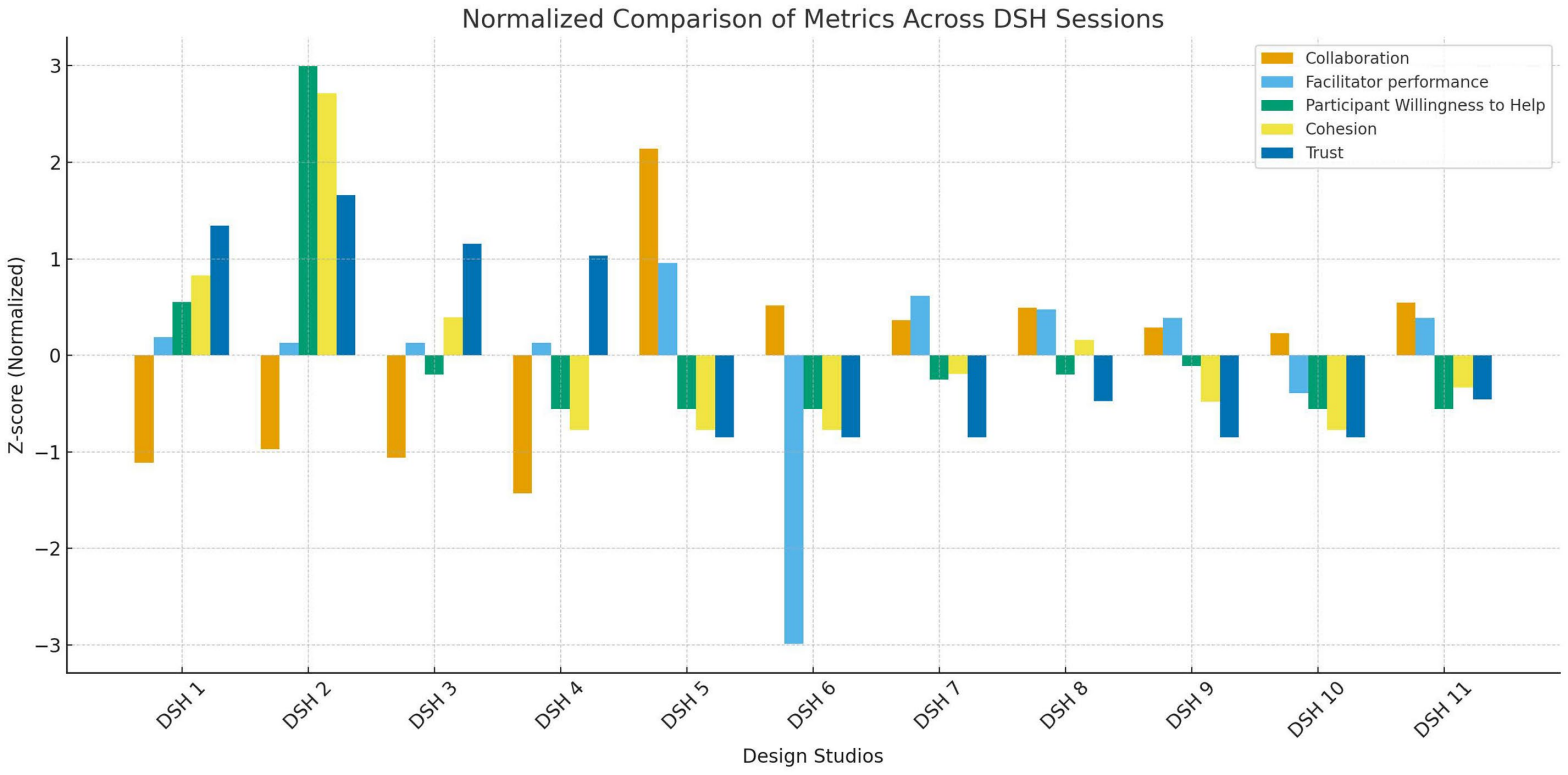
Quantitative results.

In total, 115 participants engaged across the 11 DSH sessions, and 100 completed the post-session survey with an overall response 87% rate (See Table 2). The analysis of the 11 DSH sessions reveals evolving dynamics in participant engagement and perceptions over time. Quantitative measures were obtained for five core aspects—Collaboration, Facilitator Performance, Participants’ Willingness to Help Each Other, Group Cohesion, and Trust. Early sessions (DS1–DS4) generally exhibited lower collaboration scores but higher trust levels, indicating that initial interactions, while less dynamic, established a solid foundation of confidence among participants. In contrast, later sessions (DS6–DS11) demonstrated an increase in collaborative engagement, with mean scores rising notably; however, this improvement did not extend uniformly to all aspects of group dynamics. Notably, Design Studio #5 emerged as a standout session, registering an exceptionally high collaboration score (8.8 ± 1.27) alongside the highest facilitator performance rating (1.9 ± 0.2). This suggests that specific strategies employed in DS5 may have effectively promoted teamwork and effective facilitation. Similarly, DS2 distinguished itself by achieving the highest measure for Participants’ Willingness to Help Each Other (3 ± 0) and a high level of group cohesion (4 ± 0), underscoring that targeted interventions can significantly enhance cooperative behaviors and group unity. Meanwhile, facilitator performance remained relatively stable across sessions, with scores largely ranging from 1.5 to 1.9, indicating consistency in the delivery and structure of the sessions. A particularly interesting trend is observed in the measure of trust. The early sessions reported strong trust scores—with DS1, DS2, DS3, and DS4 ranging from 4.0 to 5.0, which reflects a robust initial rapport among participants. However, as the iterative process continued in later sessions, trust scores fell markedly to levels between 1.0 and 1.62 with DS8 and DS11 showing marginally higher values (see Figure 3).

**Table 2:** Participant engagement and survey completion across DSH focus areas.

Focus area	Participants engaged (*n*)	Post-DSH session survey completed (*n*)	Response rate (%)
Community-Academic Partnership	30	25	83.3
Food Security	20	15	75
Refugee Health	20	19	95
Cancer Prevention and Control	30	28	93.3
Menopausal Women’s Brain Health	15	13	86.7
Total	115	100	87

### 3.2 Qualitative results

In addition to measurable improvements in collaboration and facilitation, qualitative insights provided rich context into participants’ lived experiences within the DSH. Participants described feeling empowered, more informed, and deeply connected to others through their engagement in the sessions. Many reported increased awareness of health issues—particularly around testing and prevention—and were inspired to share this information within their personal networks (see Table 3). A recurring theme was the importance of deep listening. Participants identified this as a cornerstone of the Design Studio process that enabled mutual understanding, trust-building, and authentic collaboration. The opportunity to engage in open, real conversations about specific, often under-addressed topics were described as both validating and transformative. Participants also consistently acknowledged the Studio’s innovative design, which created a safe and inclusive environment where diverse perspectives and life experiences were not only welcomed but centered. This environment fostered a strong sense of belonging and collective purpose, with participants frequently expressing relief and solidarity in discovering that they were not alone in their struggles. Overall, the Design Studio was recognized for fostering meaningful dialogue, community engagement, and participant empowerment—key indicators of its alignment with principles of equity, cultural relevance, and community-led change.

**Table 3:** DSH qualitative insights.

Focus area	Individuals/Community partner	DSH outcomes	Exemplar community partner quotations
Community-Academic Partnership (*N* = 30)	Community Bridges Inc., Unlimited Potential, Valley of the Sun United Way, Inter-Tribal Council of Arizona, Equality Health Foundation, CAIR, IRC, School districts	Do not co-lead with just data Conversations with community without agenda Finding together tangible health solutions Connect the communities with resources Sustain your engagement with communities	“The diversity of the attendees and the creation of a safe space… fostering inclusivity and meaningful exchanges.” “This is a very innovative approach to connect and build trust with communities.”
Food Security (*N* = 20)	Sustainable Food Center, Black and Latinx communities, and community health workers in central and west Texas	Need for representation, not tokenization Acknowledge intersectionality of race, ethnicity, and sexual orientation Need for outreach programs and opportunities for health education through cooking or workout classes	“Promoting openness and facilitating meaningful conversations and sharing” “I gained new perspectives from people who have different life events.”
Refugee Health (*N* = 20)	IRC, Valleywise Health, Mosaic Elder Refugee Program, Burundi–American Association, Sudanese Community Organization	Consistent engagement with communities for trust-building and mutual understanding Include the voice of authorities in refugee communities Promote trauma healthcare	“Very innovative approach to connect and build trust…” “Deep listening… fosters mutual understanding…” “DSH creates a safe and open meeting environment for expressing opinions and sharing ideas.”
Cancer Prevention and Control (*N* = 30)	CHS Translational Team, Black and Latinx community partners	Knowledge gain Empowerment Cultural understanding Mobile screening	“I feel so much more informed… empowered.” “Engagement of the community as the recipients in any intervention makes it more meaningful and the DSH served that purpose.”
Menopausal Women’s Brain Health (*N* = 15)	Prickly Pear Health	Balance between confidentiality and tailoring of health plans Choice to opt in or out of data sharing Transparency of data sharing	“It allowed me to share my opinions without a problem.” “I enjoyed hearing what other women think and would want from an app like this. There were some good ideas and considerations that were shared.”

## 4 Discussion

This study was designed with a twofold intent: first, to introduce and describe the Design Studios for Health framework as a novel, equity-centered model for advancing community engagement in health research; and second, to evaluate its initial implementation across 11 studios conducted in Arizona. The dual focus—model description and early evaluation reflects our goal of both contributing to the literature on equity-centered community engagement and empirically testing the feasibility of DSH as a replicable participatory framework. The findings of this study highlight the significant potential of the DSH as a novel and impactful approach to engaging communities in the co-creation of health interventions. Beyond its current applications, DSH also exemplifies how studio-based methods can be leveraged to envision future, distributed models of healthcare and community collaboration (40). By examining the performance of 11 DSH sessions across a range of health topics, this study provides important insights into how iterative, collaborative design processes can strengthen community engagement, build trust, and foster sustainable partnerships between researchers, healthcare providers, and communities. These outcomes have implications for both public health practice and the academic discourse surrounding participatory and equity-centered methodologies.

### 4.1 Contribution to practice and research

This study offers a unique contribution to the public health literature by operationalizing the design studio model as a structured, iterative framework for fostering equitable academic-community partnerships. Unlike conventional participatory methods, which often rely on one-time engagements or static consultation processes, the DSH model employs a dynamic, multi-session format grounded in design thinking principles—particularly the cycles of divergence (generating multiple ideas) and convergence (narrowing and refining them) (21, 41, 42). The model’s three-movement structure—insight identification, integration, and activation—paired with embedded feedback mechanisms (e.g., surveys, digest reports), supports continuous adaptation and deeper trust among stakeholders. Furthermore, this model demonstrates flexibility across a variety of health topics and participant populations. The application of DSH to both multi-session interventions and a standalone session (e.g., the Women’s Brain Health studio) illustrates its adaptability and scalability. In doing so, the DSH framework advances the field by merging methodologies from design and public health to more effectively engage communities in the co-production of health equity solutions, fostering the kind of transdisciplinary collaboration emphasized in contemporary design-mindset research (43).

For practitioners and researchers, DSH offers a replicable and adaptive structure for community-centered health intervention development. One key implication is the value of structuring engagement as an iterative process with multiple touchpoints. In this study, two- to three-session formats were particularly effective in building participant trust, fostering feedback loops, and enhancing solution relevance. This aligns with evidence that sustained collaboration improves intervention success and stakeholder satisfaction (44).

Trust-building emerged as a foundational component of successful studios, echoing broader public health literature on the centrality of trust in community-based work, particularly in underserved and historically marginalized populations (45). The DSH approach—emphasizing cultural humility, inclusive facilitation, and participatory structures—allowed participants to feel heard, seen, and valued, contributing to more authentic partnerships and meaningful outcomes. However, quantitative findings revealed a decline in trust scores over successive sessions. This decline highlights a potential challenge in sustaining interpersonal confidence over repeated interactions. Together, these findings indicate that while the iterative DSH process can enhance collaboration and facilitator effectiveness, additional strategies may be required to maintain and bolster trust and group cohesion as the sessions progress.

Across the 11 DSH, participant demographics reflected both gender and role diversity, with a notable predominance of women across community member and researcher groups. Female participants consistently outnumbered men, particularly among community representatives, suggesting that women may be more likely to engage in collaborative, health-related participatory processes. The academic researchers and community health organization representatives were generally older and highly educated, while community members represented a broader range of educational backgrounds, from high school to doctoral degrees. This mix enriched dialogue and co-learning but also highlighted differences in power, language, and professional familiarity that may have subtly influenced participation dynamics and trust development.

We note that DSH shares lineage with the Community Engagement Studio (CES) model, and future comparative work could more explicitly test how iterative designs compare with traditional CES in terms of stakeholder satisfaction, influence on study outcomes, and sustainability. Some constraints of CES (e.g., limited continuity and potential for superficial engagement) may be alleviated by DSH’s emphasis on repeated touchpoints and ongoing collaboration. Conversely, CES’s strength lies in its lean, focused structure and lower cost, making it a viable option for projects with limited resources. Understanding when a lighter CES model suffices versus when DSH’s deeper engagement justifies the additional investment represents a valuable direction for future inquiry.

### 4.2 Limitation

Despite its contributions, this study has several limitations. First, the relatively small sample sizes for each Design Studio limit the generalizability of findings. Although the studios engaged participants from diverse, underserved populations and covered a range of topics, the composition of each group may not reflect broader community dynamics. Future research should examine the model’s effectiveness in larger and more demographically varied settings to assess its broader applicability. Second, much of the evaluation relied on self-reported participant feedback, which can introduce bias due to social desirability or reluctance to provide negative responses. While anonymity was maintained, incorporating third-party observations or facilitator reflections could strengthen future evaluations. Third, the comparison between multi-session and single-session formats—such as the Women’s Brain Health studio—was limited due to their differing structures and goals. More systematic comparisons are needed to determine how session duration and frequency influence outcomes across different contexts. Finally, while the study captured valuable insights on facilitator performance and group cohesion, these dimensions were assessed primarily through participant perceptions. Incorporating mixed-methods evaluation including interviews, focus groups, and observational data—would yield a more nuanced understanding of the relational dynamics within DSH.

## 5 Conclusion

This study demonstrates the potential of Design Studios for Health (DSH) as an innovative and equity-centered approach to fostering community collaboration and co-creating culturally relevant health interventions. By integrating principles of design thinking with community-based participatory research, the DSH model offers a structured, iterative framework that places community voices at the center of health solution development.

The evaluation of 11 Design Studios revealed that trust-building, cultural sensitivity, and inclusive facilitation is foundational to successful engagement. The iterative, multi-session format allowed for continuous feedback and adaptation, strengthening group cohesion, facilitator effectiveness, and ultimately, the quality and relevance of co-created outcomes. Notably, the study also highlights the adaptability of the DSH approach, as shown in the successful single-session Design Studio on Women’s Brain Health, demonstrating that context-specific tailoring can still yield meaningful results. This study extends current literature by offering a hybrid model that addresses gaps in traditional participatory approaches specifically the need for creative, iterative, and design-oriented engagement methods that are also grounded in equity and mutual accountability. The DSH approach goes beyond typical consultation models by fostering sustained dialogue, co-learning, and shared ownership between communities, researchers, and practitioners. In doing so, it operationalizes values of trust, empathy, and cultural humility, which are essential for addressing persistent health inequities.

Despite initial challenges in trust-building and participation, the Design Studios ultimately served as a powerful platform for generating locally grounded, sustainable health strategies. This work underscores the importance of long-term, relationship-centered engagement and offers a practical, replicable model for community health innovation. Future research should continue to explore and refine the DSH model across diverse communities and health domains. Doing so can contribute to a deeper understanding of how participatory design processes can equitably and effectively support population health and advance community-driven change.

## Data Availability

The original contributions presented in the study are included in the article/supplementary material, further inquiries can be directed to the corresponding author.
